# A New Approach for Bioremediation of Olive Mill Wastewaters: Combination of Straw Filtration and Nanofiltration

**DOI:** 10.3390/membranes14020038

**Published:** 2024-01-31

**Authors:** Francesco Chidichimo, Maria Rita Basile, Carmela Conidi, Giovanni De Filpo, Rosanna Morelli, Alfredo Cassano

**Affiliations:** 1 Department of Environmental Engineering (DIAm), University of Calabria, Via P. Bucci 42B, 87036 Rende, Italy; francesco.chidichimo@unical.it; 2 TEBAID Consortium, Department of Chemistry and Chemical Technologies, University of Calabria, Via P. Bucci 15D, 87036 Rende, Italy; basimari88@gmail.com; 3 Institute on Membrane Technology, ITM-CNR, Via P. Bucci 17C, 87036 Rende, Italy; c.conidi@itm.cnr.it (C.C.); r.morelli@itm.cnr.it (R.M.); 4 Department of Chemistry and Chemical Technologies, University of Calabria, Via P. Bucci 15D, 87036 Rende, Italy; giovanni.defilpo@unical.it

**Keywords:** olive mill wastewaters, straw filtration, nanofiltration, phenolic compounds

## Abstract

A combination of straw filtration and nanofiltration was investigated for the first time as a sustainable approach aimed at valorizing olive mill wastewaters (OMWs) within a circular economy strategy. Ground straw filters with different granulometry (120, 250 and 500 μm) were tested in the first step to clarify the raw wastewater. The 500 μm filter offered the best performance due to a lower exposed surface of the filtering fibers and a shorter filtering time, allowing us to reduce about 70% of the chemical oxygen demand (COD) of the raw wastewater. Three different commercial membranes in a flat-sheet configuration with a molecular weight cut-off (MWCO) in the range 150–500 Da were tested to fractionate the clarified wastewater according to a dead-end configuration. Among the investigated membranes, a polymeric membrane of 500 Da (NFA-12A) exhibited the highest productivity in selected operating conditions (steady-state values of 11.4 L/m^2^ h at 20 bar and 24 ± 2 °C). In addition, flux decays for this membrane were lower than the other two tested membranes, indicating a lower propensity to fouling phenomena. Higher rejections towards total polyphenols and total antioxidant activity (TAA) (76.6% and 73.2%, respectively) were also observed for this membrane. Flavanols and hydroxycinnamic acids were retained by more than 99%. The combination of straw filtration and NF with the NFA-12A membrane allowed us to reduce the COD of raw OMWs up to 97.6%. The retentate fraction of this membrane exhibited a TAA of 18.9 ± 0.7 mM Trolox, supporting its propensity for the development of innovative formulations of interest in food and nutraceutical applications.

## 1. Introduction

The olive oil processing industry produces large amounts of liquid wastewaters, known as olive mill wastewaters (OMWs). Their disposal without any treatment generates serious environmental problems including coloring of natural waters, contamination of underground water, phytotoxicity and bad odors. The annual worldwide production of OMWs is estimated to be about 1 × 10^7^ m^3^, most of which are produced in the Mediterranean area [1].

OMWs appear as dark liquid effluents with an acid reaction. The pH, immediately after their production, is in the range 4.5 and 5.9 [2] and depends on the aging of the olives and the wastewaters due to oxidative processes catalyzed by various bacterial species. Their physico-chemical characteristics can vary widely in relation to several factors such as climatic conditions and cultivar, extraction methodology, type and maturity of olives and extraction and processing methods [3]. The pollution load of OMWs is essentially related to their high content of organic substances and phenols. The chemical oxygen demand (COD) and corresponding biochemical oxygen demand (BOD) can reach values of up 220 g L^−1^ and 100 g L^−1^, respectively [4]. Phenolic compounds, ranging from 0.5 to 24 g L^−1^, are responsible for the phytotoxicity and high resistance of OMWs to biodegradation. On the other hand, these compounds have been recognized to exhibit a wide array of biological activities such as antioxidant, free radical scavenging, anti-inflammatory, anticarcinogenic and antimicrobial activities [5]. As a consequence, they can be exploited to be used as natural additives of foodstuff for the production of dietary supplements and nutraceuticals due to their ability to provide advanced technological properties and health claims, respectively [6,7,8].

Current research trends aim at developing technological and biotechnological applications for the recovery of fine chemicals from OMWs and the production of important metabolites, respectively. This approach, in line with the principles of circular economy, aims at closing the production loop by recycling and reusing resources, bringing benefits to the environment, society and economy [9].

The high quantities of COD and BOD present in OMWs constitute a major problem, both for disposal and for the recovery of polyphenols, which would become much easier if the large percentage of suspended mucilage had been preliminarily separated. The adoption of a staged filtering process for OMW purification therefore appears very promising, using filtration methodologies which are notoriously cost-effective technologies for removing dissolved and suspended elements from water [10,11].

Membrane processes have remarkably increased over the last decades as efficient tools to improve the valorization protocols of agro-food wastewaters and by-products within a sustainable biorefinery strategy. These processes offer several advantages over conventional separation methodologies, thanks to their intrinsic properties including mild operating conditions of temperature and pressure, simple equipment, easy scale-up and scale-down, low energy consumption, non-use of chemicals and high selectivity towards specific compounds [12,13].

Membrane processes have been largely investigated in the past in order to produce effluents of acceptable quality from OMWs for safe disposal into the environment [14]. Several valorization approaches have been also proposed through the combination of membrane-based operations in a sequential design in order to recover, fractionate and concentrate phenolic compounds from these effluents [15,16,17]. Most of these studies are focused on the use of pressure-driven membrane operations such as microfiltration (MF), ultrafiltration (UF), nanofiltration (NF) and reverse osmosis (RO). In particular, MF and UF processes are mainly used as a pretreatment step to produce a clarified effluent enriched in phenolic compounds and free of suspended solids [18]. NF and RO processes are used as intermediate or final steps for purification and concentration purposes [19]. All these processes meet the requirement for the recovery, purification and concentration of polyphenols from OMWs with regard to their specific molecular weight cut-off (MWCO) values. However, the performance of MF and UF membranes is remarkably affected by fouling phenomena, which determine both an decrease in membrane productivity (permeate flux) and variation in membrane selectivity. Fouling also reduces the membrane service lifetime, making the process economically unfeasible [20,21]. In the sequential process investigated by Russo [22], based on the use of MF, UF and RO membranes, the pretreatment of raw OMWs by MF was considered a critical step of the overall process due to irreversible fouling phenomena of both polymeric and ceramic membranes investigated. Similar results were obtained by Cassano et al. [17] in the pretreatment of raw OMWs with hollow fiber UF membranes with pore size of 0.02 μm. These membranes showed higher fouling indexes (93.6%) compared with those of the UF and NF membranes (21.1 and 20.0%, respectively) used in the following steps.

Turano et al. [23] obtained a significant improvement of the permeate flux in the UF of OMWs previously submitted to a centrifuge treatment at 4000 rpm. However, the UF membranes were severely fouled by organic compounds and minerals present in OMWs even in presence of this pretreatment. Pre-treatment approaches before membrane operations based on either filtration through a 50 μm filter or flocculation eventually combined with photocatalysis and aerobic treatment have been also reported [24].

The global results confirm that the implementation of MF and UF in the pre-treatment step of OMWs is still limited by the high fouling potential of the membranes. Therefore, research efforts look at identifying tailored and cost-effective pretreatments for OMW management.

A recently patented methodology [25] for OMW purification involves the following three stages: (i) OMW acidification to a pH of 3.5 and decantation of the same for one day; (ii) water filtration, for the elimination of suspended solid particles, by using a filter made in situ by depositing a layer of straw dust (size of particles around 500 μm) above a supporting technical sheet; (iii) further filtration of the resulting permeate on a nanomembrane filter.

The possibility of using a filter made of powdered natural plant material, such as ground straw, in OMW purification processes would have the following advantages. Apart from the low cost of the starting material, generally available in the agricultural world very close to oil mills, there would be several interesting possibilities for recycling the composite made up of the filtered material and the filter itself. The composite of the two materials, having a water content in the order of 50%, could be better recycled in composting systems and directly used in the fertilization of olive groves, or, once dried, it could be tested as manure by livestock farms. An oil mill with medium production capacity would require 200 kg of straw fiber per day, approximately. This amount of fiber can be ground in one hour by means of a 10 kW power mill. The resulting power consumption (10 kWh/day) would be priced, in Europe, at 1.5 EUR. Moreover, straw fibers may hold a certain percentage of moisture, and this means that drying operations should be undertaken before grinding. The estimated power consumption and the related cost for straw drying (3 h in an oven with an electrical power of 10 kW) are of the order of 30 kWh/day and 4.5 EUR /day, respectively.

The current investigation aims at evaluating, for the first time, the performance of straw filtration as a primary treatment option in OMW purification (as an alternative to microfiltration or ultrafiltration operations) and its combination with NF as an innovative, simple and economical approach to reduce the pollution load of OMWs and, at the same time, to obtain concentrated fractions of phenolic compounds of interest for the development of nutraceutical formulations. Three commercial flat-sheet NF membranes with MWCO in the range of 150–500 Da were tested to concentrate the clarified wastewater produced after straw filtration. Their performance was evaluated in terms of productivity and selectivity towards total polyphenols, flavanols, hydroxycinnamic acid derivatives, COD and total antioxidant activity (TAA).

## 2. Materials and Methods

### 2.1. Olive Mill Wastewaters and Pre-Treatment

Olive mill wastewaters (OMWs) obtained from a 3-phase centrifugation process were supplied by Coll.J.A. Società Cooperativa, located in San Giorgio Albanese (Cosenza, Italy). Their initial pH (about 4.8) was adjusted to 3.5 by adding sulfuric acid at 98% (from Merck KGaA, Darmstadt, Germany) in order to achieve the coagulation and precipitation of suspended solids according to the Stokes law. After acidification, the raw wastewaters were submitted to decantation at room temperature for at least 24 h.

### 2.2. Straw Filtration Experiments

**Figure 1:**
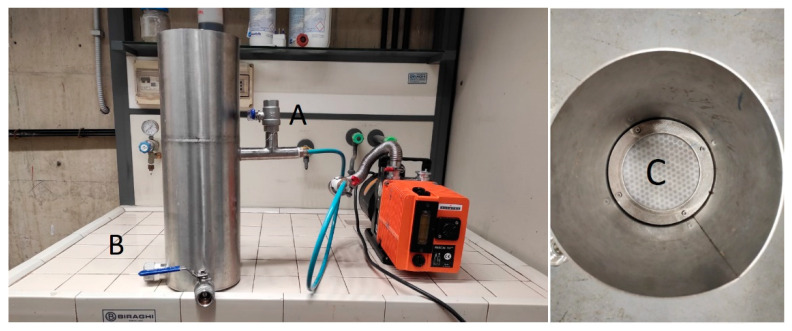
Experimental filter for the removal of suspended solids from vegetation water: (**A**) hydraulic valve connecting the lower chamber with a vacuum pump; (**B**) filtered water drain valve; (**C**) top vision of the filter with the supporting technical sheet.

The filtering apparatus consisted of a vertical stainless steel cylinder 70 cm long and 16 cm in diameter. A perforated metal plate, supporting a 120 μm pore nylon fabric, internally divided the cylinder into two equal chambers. A stainless steel funnel, housed in the lower chamber, was welded under the plate so that its terminal parts 10 cm from the bottom of the cylinder. The latter is equipped with two access points controlled by hydraulic valves: one downstream of the filtering plate for the vacuum line (Figure 1A) and the other at the bottom of the cylinder for filtered water discharge (Figure 1B).

The vacuum, in the lower part of the filtering cylinder, was created by a vacuum pump (Elnor Motors, Haacht, Belgium). Twice-ground straw, lying on the microporous nylon fabric, was used as a filtering material. A low-speed granulator (SG-1621-CE, Shini Plastics Technologies, New Tapei, Taiwan) was used to obtain a straw fine powder with a particle size of about 500 μm; a further size reduction at 250 and 120 μm was obtained by using a Retsch™ ZM 200 Model Ultra-Centrifugal mill (Retsch GmbH, Haan, Germany).

The packing of the plant-based filter was made by placing 100 g of water-soaked grinded straw on the nylon support fabric, followed by filter compaction through liquid suction. Experiments were performed by filtering the acidified and decanted vegetation water (about 1 L for each experiment) through straw filters of different granulometry (120, 250 and 500 μm).

The straw filters were previously washed with water acidified at a pH of 3.5 in order to remove possible soluble components which could be released by the filter during the filtration process. After filtration, the pH of the clarified wastewaters was adjusted to a pH of about 5.5 by adding marble powder supplied by local marble factories.

### 2.3. Nanofiltration Experiments

Nanofiltration (NF) experiments were performed according to a dead-end configuration by using a Sterlitech TM HP 4750 high-pressure stirred cell (Sterlitech, Kent, WA, USA) with a filter area of 13.85 cm^2^ and a processing capacity of 300 mL. A nitrogen cylinder, equipped with a two-stage pressure regulator, was connected to the top of the stirred cell to supply the desired pressure for filtration experiments. The cell filtration system was equipped with flat-sheet membranes, whose characteristics are reported in Table 1. The experiments were performed at an applied pressure of 20 bar and an operating temperature of 24 ± 2 °C. Stirring inside the cell was accomplished by using a magnetic stirrer. An initial volume of filtered OMW of 150 mL was used and the permeate was collected separately up to a final volume of 50 mL, corresponding to a volume concentration factor (VCF) of 3.

The permeate flux (*J*), expressed as L/m^2^h, was determined by measuring the permeate volume collected in a given time according to Equation (1):

(1) $$J=\frac{V_{p}}{t\cdot A}$$

where *V_p_* (L) is the permeate volume, *t* (h) is the permeation time and *A* (m^2^) is the membrane area. Normalized flux was determined by dividing the flux at a specific time (*J*) by the initial flux (*J*_0_), providing the percentage of the original flux that was preserved.

The rejection rate (*R*) of a specific compound was calculated as described in Equation (2):

(2) $$R=(1-\frac{C_{p}}{C_{f}})\cdot 100$$

where *C_p_* and *C_f_* refer to the solute concentration in permeate and feed samples, respectively.

After the treatment with the clarified wastewaters, the selected membranes were washed with water for 15 min and then submitted to cleaning with an enzymatic solution (P3-Ultrasil 53, 1 wt%, ECOLAB-Henkel) at 40 °C for 60 min. After that, the membranes were rinsed with distilled water for 15 min and the hydraulic permeability was measured once again.

**Figure 2:**
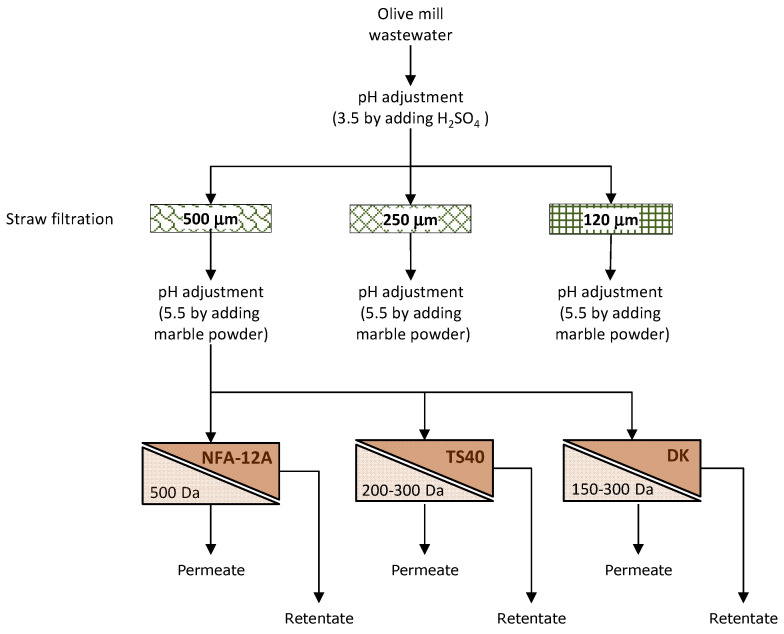
Block diagram of experimental activities.

The block diagram of the investigated process is depicted in Figure 2.

### 2.4. Analytical Determinations

#### 2.4.1. Total Soluble Solids, pH and Electrical Conductivity

The total soluble solids (TSS) were measured by using a hand refractometer (Atago Co., Ltd., Tokyo, Japan) with a scale range of 0–32 °Brix. pH was measured by an Orion Expandable ion analyzer EA 920 pH meter (Allometrics, Inc., Baton Rouge, LA, USA) with automatic temperature compensation. The electrical conductivity was measured by a Five Easy FE30 conductivity meter (Mettler-Toledo S.p.A., Milano, Italia).

#### 2.4.2. Total Polyphenols

The total polyphenols were estimated colorimetrically using the Folin–Ciocalteu method [29]. The method is based on the reduction in tungstate and/or molybdate in the Folin–Ciocalteu reagent byphenols in an alkaline medium, resulting in a blue-colored product. Gallic acid was used as a calibration standard and the results were expressed as the gallic acid equivalent (mgGAE/L). The absorbance was measured using a UV-visible spectrophotometer (ShimadzuUV-160A, Kyoto, Japan) at 765 nm.

#### 2.4.3. Total Antioxidant Activity

The total antioxidant activity (TAA) was determined according to an improved version of the 2,2-azino-bis-(3-ethylbenzothiazoline-6-sulphonic acid (ABTS) radical cation decolorization assay, in which the radical monocation (ABTS^+^) is generated by the oxidation of ABTS (Sigma Aldrich, Milano, Italy) with potassium persulphate (Sigma Aldrich, Milano, Italy) before the addition of the antioxidant [30]. The results were expressed as trolox equivalent antioxidant capacity (TEAC).

#### 2.4.4. Flavanols and Hydroxycinnamic Acid Derivatives

Flavanols and hydroxycinnamic acid derivatives were determined according to the method reported by Obied et al. [31]. An amount of 1 mL of diluted ethanolic extract (1:10 in water) was mixed with 1 mL of HCl-ethanol solution (0.1 mL HCl/100 mL in 95 mL ethanol/100 mL) into a 10 mL volumetric flask and the volume was made up to 10 mL with 2 mL HCl/100 mL. After mixing, the absorbance was measured at 320 and 360 nm to determine hydroxycinnamic acid derivatives and flavanols, respectively. The results were expressed as mg/L of caffeic acid (λ, 320 nm) and quercetin (λ, 360 nm), respectively.

#### 2.4.5. Chemical Oxygen Demand

Chemical oxygen demand (COD) measurements were carried out using a photometric test kit (COD Cell Test C4/25). Spectrophotometric measurements were performed by using a PhotoLab 7600 UV-VIS—WTW spectrophotometer (Xylem Analytics Germany Sales GmbH & Co. KG., Weilheim, Germany).

#### 2.4.6. Statistical Analysis

All analytical measurements were conducted in triplicates and the results were expressed as the average of the three measures ± the standard deviation.

## 3. Results and Discussion

### 3.1. Straw Filtration

**Figure 3:**
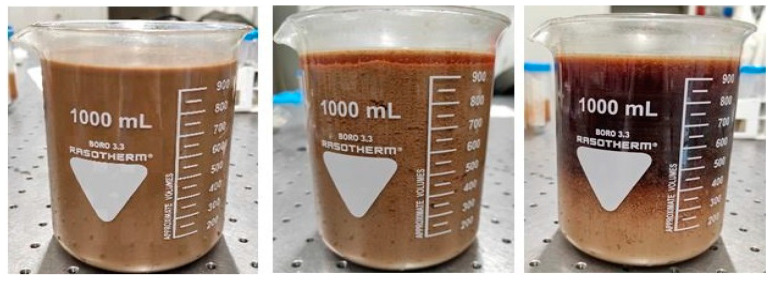
From left to right: vegetation water as it is, acidified vegetation water after 1 h and then after 24 h.

After acidification with sulfuric acid, the raw wastewater was left to flocculate/settle so that solid micro-particles could merge and form larger particles which were easy to be separated from the liquid phase. According to Bazzarelli et al. [32], the zeta potential of the OMWs at pH 4.5 is −30 mV, resulting in greater electrostatic repulsion forces between particles and reduced aggregation/flocculation phenomena caused by Van der Waals interactions. The pH adjustment destabilizes the suspension, reducing the zeta potential of the particles (absolute value) and promoting their aggregation and flocculation. Following the acidification process, the vegetation water was split into two distinct phases: a darker and clearer upper phase and a lighter and cloudier lower phase, in which the solid organic material, at first homogeneously distributed throughout the volume, is settled (Figure 3).

**Figure 4:**
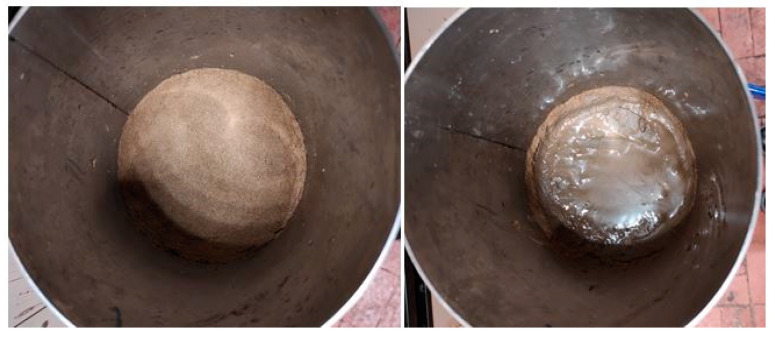
Clean straw filter (**left** picture); solid residues on the filter surface (**right** picture).

As reported previously, the decanted water was filtered through straw powder filters of different granulometry: 120, 250 and 500 μm. After filtration, about 100 mL of the saturated solid residues were retained by the filter. Figure 4 shows the 3.5 cm layer of powdered plant material supported by the nylon fabric, on the left, while the gel retained by the filter, above the aforementioned layer, is visible on the right.

The straw filters showed an excellent purifying capacity; however, the filtration rate decreased over time due to the clogging effect of the slime film, gradually deposited by the liquid flow on the filter surface. Anyway, the filtering capacity was easily restored without affecting the thickness of the purifying layer, whose intrinsic porosity did not change, by simply removing the shallow jelly film mechanically. Such an operation allowed the 500 μm filter to maintain a filtering capacity of 0.8 m^3^/m^2^h. If the slime derived from the vegetation water is mixed with a similar weight of the plant material adopted for the filter, a friable and easily compostable composite could be obtained. This composite material, in fact, unlike the filtered one with a compact jelly structure, is highly permeable by air. We believe that this is one of the strengths of the developed filtration system. The gelatinous solids, gradually removed from the filter, can be actually delivered, together with the filtering material, to a suitable composter for the production of agricultural amendment.

The filtered water produced in each test (900 mL) was then brought back around the original pH, by adding marble powder (CaCO_3_), before the NF tests. The COD was measured again after pH balancing. The results of the straw filtration experiments are summarized in Table 2. The filtration rate with the 120 μm filter was 80% lower than that obtained with the 500 μm filter (23.7 mL/min against 112.5 mL/min). The removal of COD for the straw filters investigated was in the range of 64.8–73.3%, with the 500 μm filter exhibiting the highest removal percentage (73.3%).

**Figure 5:**
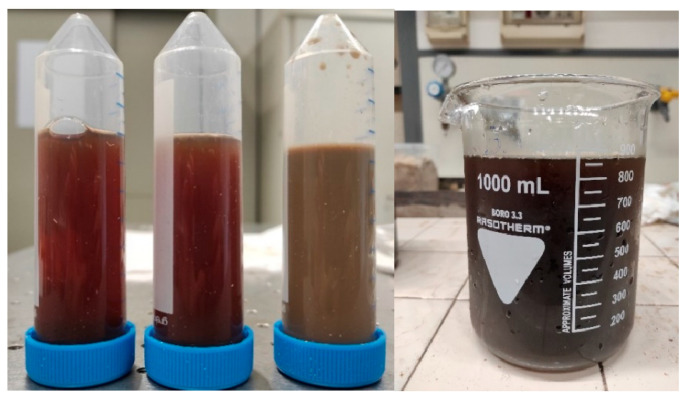
The left picture shows samples S120, S500 and US collected for COD measurement; in the right picture is the vegetation water filtered and neutralized at pH 5.5.

The observed differences in the water filtered using different size straw filters are slightly higher with respect to the measurement experimental errors. They must be probably attributed to the small quantities of dissolved straw components, which were not completely removed during the washing of the powder. A different small contribution of these soluble components is in fact expected in the various experiments due the different filtering size particles and different filtering times. These considerations could justify the best result obtained in the case of the 500 μm filter due to a lower exposed surface of the filtering fibers and to a shorter filtering time. The neutralized waters presented a bright black color, as shown in the picture on the right in Figure 5.

### 3.2. Nanofiltration

**Figure 6:**
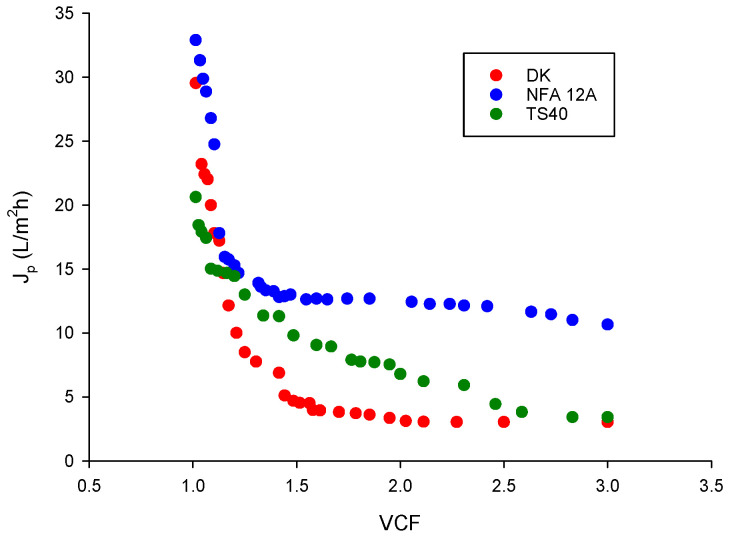
Nanofiltration of clarified OMWs. Permeate flux as a function of VCF for selected membranes. Operating conditions: applied pressure, 20 bar; temperature, 24 ± 2 °C.

Clarified wastewaters coming from the filtration step with straw powder ground at a size of 500 μm and neutralized at pH 5.5 were submitted to an NF treatment with three different flat-sheet membranes in selected operating conditions (applied pressure, 20 bar; temperature, 24 ± 2 °C). Figure 6 shows the evolution of permeate flux in terms of liters of permeate produced per unit area and time (L/m^2^h) as a function of VCF for the investigated membranes. For all membranes, the J_p_ versus VCF curve can be divided into three periods: an initial period in which a rapid decrease in permeate flux occurred; a second period, up to VCF 2, corresponding to a smaller decrease in permeate flux; a third period, characterized by a small decrease in permeate flux up to a steady-state value.

It is known that flux decline can be caused by various factors such as concentration polarization, gel layer formation and pore blocking by OMW components. All these factors produce further resistances on the feed side to transport across the membrane [33].

The NFA-12A membrane exhibited a better performance in terms of initial (32.9 L/m^2^ h) and steady-state (11.4 L/m^2^ h) permeate fluxes in comparison with the other tested membranes. This result can be attributed to the higher MWCO of this membrane, as well as to its higher hydrophilicity. In particular, the permeate flux values were similar to those reported by Zirehpour et al. [33] for spiral-wound NF membranes (NF-270 and NF-90 membranes with MWCO of 200–250 and 150–200 Da, respectively) in the treatment of microfiltered OMWs. Similarly, Bazzarelli et al. [32] reported permeate flux values lower than 10 L/m^2^h in the treatment of microfiltered OMWs with a spiral-wound NF-90 membrane (at an operating pressure of 10 bar) with an MWCO of 200 Da. Therefore, the global findings clearly show that the straw filtration operation is able to reduce submicron particles of OMWs, producing clarified solutions that meet the SDI index to be concentrated with commercial spiral-wound NF membranes.

**Figure 7:**
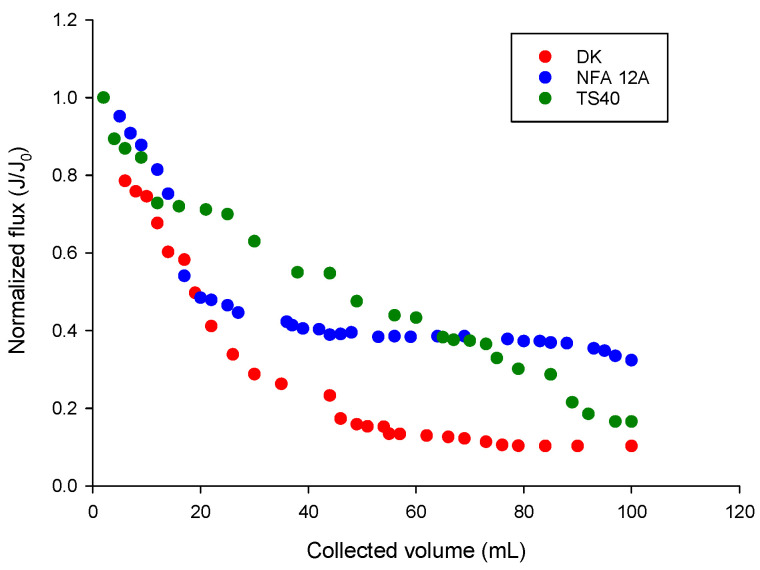
Nanofiltration of clarified OMWs. Evolution of the normalized permeate flux with cumulative permeate volume. Experimental conditions: applied pressure, 20 bar; temperature, 24 ± 2 °C.

The normalized flux as a function of the collected permeate volume (Figure 7) also showed a steep decrease for all selected membranes: however, flux losses were around 67.6% of the original value for the NFA-12A membrane, whilst flux losses for membranes TS40 and DK were 83.4% and 89.7%, respectively.

These results confirm a lower propensity of the NFA-12A membrane to be fouled as a consequence of its higher hydrophilicity. In addition, the enzymatic washing allowed the measured water permeability to be fully recovered for the pristine membrane.

Analytical measurements performed on the feed, permeate and retentate samples treated with the investigated membranes are reported in Table 3, Table 4 and Table 5. For all selected membranes, the TSS content of the permeate was about 1.0–1.2 °Brix, while the electrical conductivity was in the range of 18.7–19.6 mS/cm. Accordingly, the reductions in these parameters, in comparison to the clarified wastewater, were of the order of 64–69% and 47–49%, respectively.

**Figure 8:**
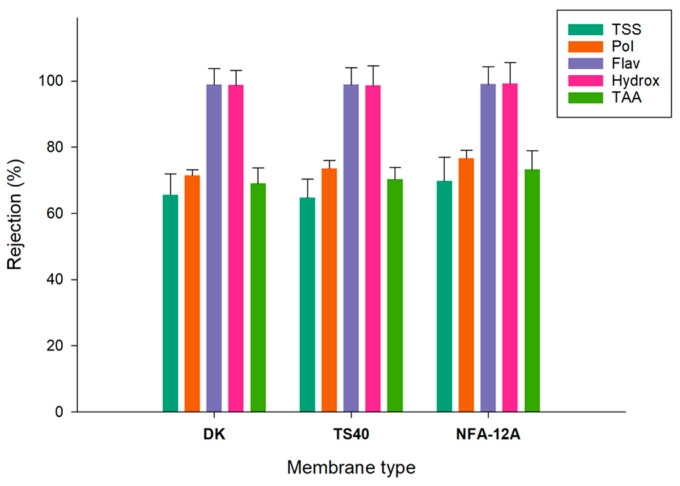
Rejections of NF membranes towards specific compounds (TSS, Total Soluble Solids; Pol, total Polyphenols; Flav, Flavanols; Hydroxy, Hydroxycinnamic acid derivatives; TAA, Total Antioxidant Activity).

The rejections of the selected membranes towards the analyzed compounds are illustrated in Figure 8. For all membranes, the rejections towards flavanols and hydroxycinnamic acid derivatives were of the order of 99%. Similar results were obtained by Conidi et al. [34] in the treatment of clarified olive mill solid-waste aqueous extracts with polymeric membranes with an MWCO in the range of 150–500 Da, including the NFA-12A membrane.

Rejections for total phenolic compounds were in the range of 71.5–76.6%, with the NFA-12A membrane exhibiting the highest value (76.6%). These values are higher than those measured by Alfano et al. [35] in the treatment of OMWs based on an initial flocculation with celite, followed by a sequential treatment with ultra- and nano-filtration spiral-wound membranes in polyethersulfone with an MWCO of 100 kDa and 3–5 Da, respectively. Specifically, the NF permeate showed a reduction of about 95% of the organic load and the polyphenol recovery, after two filtration steps, was about 65% *w*/*v*.

The rejection for TAA was between 69% and 73.2%, in agreement with the rejection observed for the total polyphenols. As a result, the retentate fraction obtained with the NFA-12A membrane presented the highest concentration of phenolic compounds (3785.1 ± 20.3 mg GAE/L), as well as the highest value of TAA (18.9 ± 0.7 mM Trolox). These data confirm previous findings about the correlation between the content of total phenols measured with the Folin–Ciocalteu method and the antioxidant activity, determined by different methods including ABTS and DPPH assays [36]. On the other hand, the DK membrane, with the lowest MWCO (150–300 Da), showed a lower rejection towards phenolic compounds in comparison with the other two NF membranes. These results confirm previous findings regarding the non-strict correlation between the MWCO and separation of phenolic compounds on the basis of their molecular weight. Indeed, other factors apart from steric mechanisms, including the hydrophobicity of the membrane surface and the solubility of the solutes, can affect the separation mechanism, reducing the impact of the pores’ dimension [37]. The interaction between phenolic compounds and/or retained macromolecules to form large-sized particles which can be adsorbed on the membrane surface can also contribute to increasing the resistance to permeation [38].

**Figure 9:**
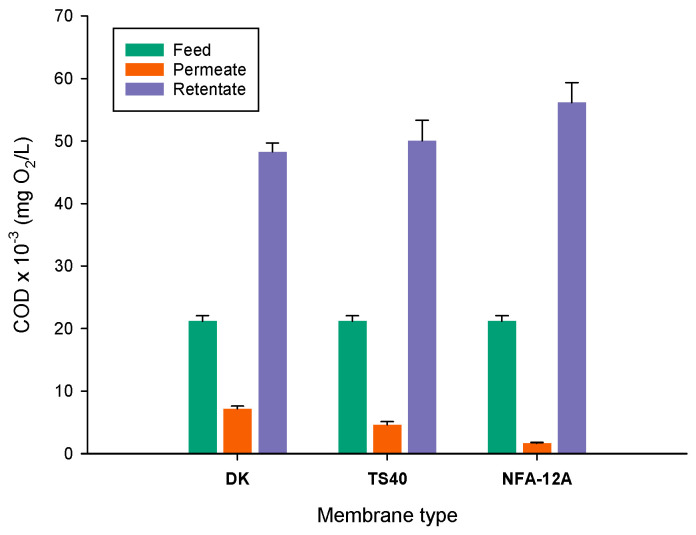
COD evaluation in samples from NF of clarified OMWs with different membranes.

The COD evaluation in samples from NF of clarified OMWs with different membranes is illustrated in Figure 9. According to the measurements in permeate samples, the reduction in COD in the clarified wastewaters with the DK, TS40 and NFA-12A membranes was 66.1%, 78.2% and 92.1%, respectively. These data are also in agreement with those observed for total phenol rejections, with the NFA-12A membrane exhibiting the highest rejection value.

The global results presented herein demonstrated that the combination of straw filtration and NF with the NFA-12A membrane effectively reduces more than 97% of the initial COD of raw wastewaters, with a final COD in the NF permeate of 1668 mg/L. These results are particularly encouraging if we take into account the simpler treatment of vegetation water compared to those proposed so far. For example, COD reductions of 94% were observed by Coskun et al. [39] in the pre-treatment of OMWs by centrifugation followed by ultrafiltration and NF (with an NF-270 membrane with an MWCO of 200–300 Da). The final COD measured in the NF permeate resulted in 5800 mg/L. The use of RO at 25 bar pressure, instead of NF, improved the removal of COD with one production of a permeate having a COD of 1000 mg/L. In another approach, the combination of lime precipitation, filtration with a membrane filter press and filtrate post-treatment using activated carbon adsorption produced a maximum removal of total organics of 80% [40]. Other proposed processes allow a complete reclamation of OMWs without considering the recovery of phenolic compounds. For instance, in the approach proposed by Ochando-Pulido et al. [41], the COD of olive mill effluents from a two-phase extraction process was reduced up to 99.1% through a depuration procedure integrating both an advanced oxidation process based on Fenton’s reagent (secondary treatment) coupled with a final reverse osmosis (RO) stage (tertiary or purification step).

## 4. Conclusions

An eco-sustainable process is presented to valorize olive mill wastewaters for the recovery of natural antioxidants in a logic of the circular economy. In this approach, straw filtration was investigated for the first time as a pretreatment step to produce a clarified effluent containing most phenolic compounds. The solids removed from the filter, together with the filtering material, represent a suitable composter for the production of agricultural amendments.

Among the ground straw filters investigated, the 500 μm filter offered the best performance in terms of filtering time and removal of organic substances, with a reduction of more than 70% of the organic load of the raw wastewater.

Three NF membranes in flat-sheet configuration were tested to recover and concentrate bioactive compounds from the clarified wastewaters. Among the investigated membranes, the NFA-12A membrane showed the highest productivity and the lowest propensity to membrane fouling in the selected operating conditions. This membrane rejected more than 99% of the flavanols and hydroxycinnamic acids of clarified wastewaters. Rejections towards total polyphenols and TAA (76.6% and 73.2%, respectively) were also higher than those observed for the other membranes. As result, the NF retentate fractions appear of practical interest for the production of food additives and food supplements, as well as of cosmeceuticals, due to their high content of phenolic compounds.

The permeate of the NF showed COD values of about 1.6 g O_2_/L, with a reduction of about 97.6% of the organic load when compared to the initial wastewater. This purified fraction could be reused for irrigation purposes, membrane cleaning and washing of manufacturing equipment in the olive oil industry.

The desired target requirement in terms of the COD, which is below 500 mg/L, according to the Italian allowance for the municipal sewer system discharge of wastewaters, could be reached by treating the NF permeate through an additional RO step.

## Figures and Tables

**Table 1:** Characteristics of NF flat-sheet membranes (PA-TFC, polyamide–thin film composite; MWCO, molecular weight cut-off).

Membrane type	NFA-12A	TS40	DK
Manufacturer	Parker	Microdyn-Nadir	GE Osmonics
Membrane material	PA-TFC	PA-TFC	PA-TFC
Configuration	flat-sheet	flat-sheet	flat-sheet
Nominal MWCO (Da)	500	200–300	150–300
pH operating range	3–11	1–12	3–9
Max. operating temperature (°C)	63	50	50
Max. operating pressure (bar)	30.6	41	41
Contact angle (°)	10 ^a^	30 ^b^	41 ^c^
Water permeability at 18 ± 1 °C (L/m^2^ h bar)	9.2 ^d^	8.60 ^d^	3.64 ^d^

Data from ^a^ [26]; ^b^ [27]; ^c^ [28]; ^d^ own measurement.

**Table 2:** Results of straw filtration experiments on OMW samples.

ID	Description	Filtration Rate (mL/min)	Solid Residues (mL)	COD (mg O_2_/L)	pH
US	OMW unaltered state	-	-	70,000 ± 3500	4.80 ± 0.24
S120	Acidified OMW filtered with 120 μm straw	23.7	~100	22,300 ± 1115	3.5 ± 0.17
S250	Acidified OMW filtered with 250 μm straw	69.2	~100	24,650 ± 1232	3.5 ± 0.16
S500	Acidified OMW filtered with 500 μm straw	112.5	~100	18,700 ± 935	3.5 ± 0.17
S120N	OMW filtered with 120 μm straw + marble powder	-	-	22,500 ± 1125	5.5 ± 0.27
S250N	OMW filtered with 250 μm straw + marble powder	-	-	23,350 ± 1167	5.5 ± 0.27
S500N	OMWW filtered with 500 μm straw + marble powder	-	-	21,200 ± 1060	5.5 ± 0.27

**Table 3:** Physico-chemical characteristics of feed, permeate and retentate samples from the treatment of OMWs with NFA-12A membrane.

Parameter	Feed	Permeate	Retentate
TSS (°Brix)	3.3 ± 0.1	1.0 ± 0.1	7.3 ± 0.1
Electrical conductivity (mS/cm)	36.7 ± 0.7	18.7 ± 0.4	52.8 ± 0.6
Total polyphenols (mg GAE/L)	1918.4 ± 30.2	449.0 ± 12.7	3785.1 ± 20.3
Flavanols (mg/L quercetin)	623.53 ± 24.95	6.03 ± 0.22	1564.70 ± 66.55
Hydroxycinnamic acid derivatives (mg/L caffeic acid)	445.2 ± 17.4	3.49 ± 0.18	1186.3 ± 23.24
TAA (mM Trolox)	10.1 ± 0.7	2.7 ± 0.1	18.9 ± 0.7

**Table 4:** Physico-chemical characteristics of feed, permeate and retentate samples from the treatment of OMWs with TS40 membrane.

Parameter	Feed	Permeate	Retentate
TSS (°Brix)	3.4 ± 0.1	1.2 ± 0.1	5.6 ± 0.5
Electrical conductivity (mS/cm)	38.1 ± 0.3	19.6 ± 0.2	47.8 ± 0.7
Total polyphenols (mg GAE/L)	1973.3 ± 40.6	520.4 ± 12.7	3223.5 ± 71.2
Flavanols (mg/L quercetin)	605.88 ± 24.95	6.61 ± 0.21	1341.17 ± 58.23
Hydroxycinnamic acid derivatives (mg/L caffeic acid)	426.02 ± 23.24	5.96 ± 0.17	1006.85 ± 48.43
TAA (mM Trolox)	10.1 ± 0.4	3.0 ± 0.1	17.6 ± 1.1

**Table 5:** Physico-chemical characteristics of feed, permeate and retentate samples from the treatment of OMWs with DK membrane.

Parameter	Feed	Permeate	Retentate
TSS (°Brix)	3.2 ± 0.1	1.1 ± 0.1	5.7 ± 0.1
Electrical conductivity (mS/cm)	37.2 ± 0.2	19.4 ± 0.1	45.3 ± 1.2
Total polyphenols (mg GAE/L)	1960.8 ± 40.0	559.2 ± 7.1	3505.9 ± 42.3
Flavanols (mg/L quercetin)	611.76 ± 22.3	6.91 ± 0.22	1458.82± 58.23
Hydroxycinnamic acid derivatives (mg/L caffeic acid)	436.98 ± 13.56	5.34 ± 0.18	1173.97 ± 32.93
TAA (mM Trolox)	10.0 ± 0.6	3.1 ± 0.1	18.4 ± 0.6

## Data Availability

Data are contained in this article.
